# Automatic Museum Audio Guide

**DOI:** 10.3390/s20030779

**Published:** 2020-01-31

**Authors:** Noelia Vallez, Stephan Krauss, Jose Luis Espinosa-Aranda, Alain Pagani, Kasra Seirafi, Oscar Deniz

**Affiliations:** 1 Visilab (Vision and Artificial Intelligence Group), University of Castilla-La Mancha (UCLM), E.T.S.I. Industrial, Avda Camilo Jose Cela s/n, 13071 Ciudad Real, Spain; JoseL.Espinosa@uclm.es; 2 DFKI (Deutsches Forschungszentrum für Künstliche Intelligenz), Augmented Vision Research Group, Tripstaddterstr. 122, 67663 Kaiserslautern, Germany; stephan.krauss@dfki.de (S.K.); alain.pagani@dfki.de (A.P.); 3 Fluxguide, Burggasse 7-9/9, 1070 Vienna, Austria; kasra@fluxguide.com

**Keywords:** internet of things (IoT), computer vision, automatic audioguide, artificial intelligence, systems on chip (SoC)

## Abstract

An automatic “museum audio guide” is presented as a new type of audio guide for museums. The device consists of a headset equipped with a camera that captures exhibit pictures and the eyes of things computer vision device (EoT). The EoT board is capable of recognizing artworks using features from accelerated segment test (FAST) keypoints and a random forest classifier, and is able to be used for an entire day without the need to recharge the batteries. In addition, an application logic has been implemented, which allows for a special highly-efficient behavior upon recognition of the painting. Two different use case scenarios have been implemented. The main testing was performed with a piloting phase in a real world museum. Results show that the system keeps its promises regarding its main benefit, which is simplicity of use and the user’s preference of the proposed system over traditional audioguides.

## 1. Introduction

The paradigm of cyber-physical systems (CPSs) aims at a major intertwining of both the computing system and the physical world [1]. Note, however, that we humans are the best example of such intertwining and adaptation. This means that, apart from “classic” (yet still necessary) datalogging and control capabilities, CPSs have to embed intelligence. An analysis made on a number of sectors has confirmed that the key selling features of many products are now determined by their embedded intelligence [2]. In turn, human intelligent behavior crucially rests on (human) perception capabilities. In this respect, the amount of information we infer from images alone is impressive. It is estimated that eyes are responsible for 80% of the information that our brain receives. Furthermore, given the amount of data generated by our visual processing system, we rely on subconscious information processing to apply selective attention processing and extract relevant meaning from the data to quickly access and act in situations. Such decision making is made “instinctively” with our consciousness barely informed. Therefore, to build intelligent systems we must be able to replicate similar capabilities with sensors together with advanced visual processing capabilities.

Computer vision is a discipline of artificial intelligence that aims at inferring useful information from images [3]. Coupled with advances in machine learning and massively parallel processing, the possibilities of vision are endless. Computer vision is a mature research field from a theoretical point of view, although practical ubiquitous vision has not progressed comparably. In the scientific and technical literature, the closest systems to an “eyes everywhere" paradigm are to be found under the terms “embedded vision" and “vision sensor networks". On the one hand, embedded vision refers to vision systems that are integrated into more complex devices such as automotive or medical equipment. Those systems are a natural evolution of fixed industrial vision systems based on PCs and smart cameras [4]. While such systems are increasingly used, power and size requirements are not so stringent, development is typically application-specific, and connectivity is not generally an issue [5,6]. Required speed is typically achieved through the use of reconfigurable hardware [7], which limits flexibility in terms of development platforms.

Vision sensor networks, on the contrary, aim at smaller power-efficient vision sensor nodes that can operate in a standalone fashion. This is a natural evolution of the significant research effort made in the last years on wireless sensor networks (WSN). Research on vision WSNs (VWSN) has been significant (see surveys [8,9,10,11,12,13]. Platforms can be roughly divided into two groups:

- Computer vision platforms, i.e., no video streaming. Examples: [14,15,16,17].
- Streaming platform, with computer vision used to improve compression or stream only on interesting events. These have been also called ‘multimedia wireless sensor networks’. Examples: [18,19].

Platforms for VWSNs have been typically demonstrated in a specific application (surveillance, tracking, etc.). They are generally assessed in terms of energy consumption and the complexity of the demonstrated computer vision task. While these aspects are obviously important, a development platform requires attention to other aspects such as the operating system, programming, vision libraries, and interoperability with existing networks. As a consequence, existing VWSN platforms are mostly research-level prototypes that use non-mainstream network technologies (6LoWPAN, ZigBee, IEEE 802.15.4, etc.) and exotic operating systems, languages, and tool chains (TinyOS, Contiki, nesC, etc.). This has prevented widespread adoption and commercialization, which has been recently observed by some researchers [20]: “Given the fair number of proposed designs, it is somewhat surprising that a general-purpose embedded smart camera, based on an open architecture is difficult to find, and even more difficult to buy”.

Interestingly, mobile devices such as smartphones and tablets are the closest current example to versatile mobile vision systems. Driven by consumer demand, these devices currently have three key elements: Impressive computational power, connectivity, and imaging capabilities. Not surprisingly, the computer vision community has found a highly versatile platform in smartphones and tablets. Pre-existing CV and augmented reality libraries and applications are being ported to such devices and, most importantly, novel applications are arising. Arguably, this is the closest computer vision has ever come to be in the hands of everyone. Advances in mobile devices’ capabilities have overshadowed those of vision platforms developed in the context of WSN research. Despite the phenomenal success around mobile devices and their imaging capabilities, there are, however, some factors that are crucial: (a) Power and (b) unused sensors. To illustrate the problem with power, take for example one of the visual application fields of mobile devices: Augmented reality (AR). AR makes use of highest-bandwidth input sensor, i.e., video cameras. Combined with computer vision algorithms, AR has the potential to provide content that actually alters our visual reality. However, the biggest problem with providing AR that meets a user’s expectations is power. AR at 25–30 Hz that seamlessly integrates content from multiple sources requires power that is simply not available in current mobile devices. Besides, a single RGB camera with a few megapixels is no longer expected, as we soon will see hundreds of megapixels, embedded stereo pairs, HDR, embedded depth cameras, etc. All of these devices increase power requirements.

On the other hand, since reality is never switched-off, one could argue that AR should be designed to be “always-on”, which in practice would require continuous operation for hours or even days on end. Again, power is of utmost importance. Current smartphone application processors like the state-of-the-art Qualcomm Snapdragon [21] take 5–6 W to run 30 fps AR at video graphics array (VGA) resolution using all of the available processing power. Given the limitations in terms of battery technology and thermal dissipation, this means that AR applications can run for short bursts for a total time of perhaps 30 min, which is acceptable for demonstrations but is of little practical use. In practice, peak power will have to be reduced by at least one order of magnitude in order to make for a commercially usable AR solution. The mobile device industry is forced to accommodate new processors, sensors, and high resolution screens within a relatively fixed power-budget of 4–6 W. The reason for this hard limit is that the thermal dissipation capacity for handheld devices is very limited and devices can become literally “too hot to handle” if they are exceeded for any length of time, and the scenario is even worse in wearable devices where not only temperature and battery capacity but also weight and physical dimensions are also hard limits. Although modern smartphone processors can be leveraged for computer vision tasks, smartphone cameras (and Apps for them) are not intended to always be on. Context-aware applications are consequently out of the equation and most of the time we find that our mobile devices have unused sensors on board. Apart from the “always-on” capability, vision systems have been traditionally difficult to deploy. Classic applications of computer vision such as factory automation and inspection, sizes/weights, cost, and software distribution and configuration, have not been as important as other factors (such as precision and speed).

In the context described above, the eyes of things (EoT) project designed a general-purpose small embedded device for computer vision that includes the Intel Movidius Myriad2 processor. The entire project was based on four fundamental pillars: Cost, power consumption, size, and programming flexibility [22,23]. This platform is optimized to maximize inferred information per milliwatt and adapt the quality of inferred results to each particular application. This not only means more hours of continuous operation, but also the possibility of creating novel applications and services that were previously unfeasible due to size, cost, and power restrictions. In this paper, we describe such an experience, in which EoT is used to develop a novel automatic “museum audio guide”, where an audio headset is equipped with the highly-efficient EoT board and a small camera. This provides a new wearable embedded device able to recognize pictures automatically and provide audio feedback in museums. For the first time, we demonstrate that its efficiency and power consumption allows it to perform an advanced function continuously during the museum’s opening hours without recharge. The system was satisfactorily evaluated in a test pilot at the world-famous Albertina Museum in Vienna.

This paper is organized as follows. Section 2 reviews the literature on this area. Section 3 describes the hardware needed and the design of the museum audio guide headset prototype. Section 4 and Section 5 explain the use case scenarios and the applications that have been developed. The way in which paintings are recognized is explained in Section 6. Finally, Section 7 shows the information related to the test pilot performed and the evaluation results of the system and Section 8 summarizes the conclusions.

## 2. Related Work

Classic audio guide tours are based on points of interest (POIs) or stops, like key exhibits. For each stop an audio track is available. To access and playback audio tracks, in most cases, the numbers are physically situated close to the exhibits. The drawback of such solutions is (1) that a physical intervention is needed in the exhibition space (number labels), and (2) that the visitor has to carry a device permanently in his/her hands or around the neck. Moreover, ongoing user interaction with the device is necessary which may disrupt the visit and distract from the museum experience [24].

Several studies have analyzed and proposed methods to enhance the tourist’s experience in several ways automatically, without the requirement of a guide [25].

In [26], a solution based on a mobile context-aware tour guide was proposed, combining the user’s current location with the history of past locations, providing a service similar to a real tour guide in both indoor and outdoor situations. A similar approach was followed by [27]. In this case, the position is also required but the tourists use a combination of hand-held and wearable devices to obtain the information associated with the tour. This approach tries to reduce the interaction between the system and tourists sharing the tour context between the hand-held and wearable devices, guiding the users by compensating the individual weaknesses of the devices. In line with this idea, [28] proposed an audio augmented reality system to recreate the soundscape of a medieval archaeological site to enhance the user experience by improving the immersion of the participants and [29] provided augmented reality (AR) and additional information about buildings affected by earthquakes in a certain city. [30] also employed AR combined with maps to enrich the user’s experience. In [31] the user’s gaze direction information was added to outdoor positioning to include the user’s point of view. Finally, [32] discarded AR and used only positioning to keep the environment in focus and reduce distractions.

Recently, a different approach based on visual recognition instead of positioning has being considered to provide museum guides with the capability of providing information in real time. The elements to be recognized are either the exhibits or some kind of code associated with them. In [33] the scene was recognized for tourist information access using discriminative patches and invariant local descriptors extracted from the image captured by the smartphone camera. [34,35] also extracted image keypoints to retrieve the available information. In [36], the authors explored the performance of eigenpaintings, speeded-up robust features (SURF) and the combination of both methods using a mobile device to capture the images. The results showed how several aspects like reflections, changing light conditions and perspective variations may affect results. In addition, the natural markers of the artwork can be used to recognize the paintings as demonstrated in [34,35,37], with [37] including an augmented reality application to improve the experience of the user using a portable device. Mixed solutions combining positioning and image recognition can also be found [38]. On the other hand, in [39,40], “artcodes” were scanned instead with the same objective. However, [41] provided an extensive analysis of the untapped potential that this type of mobile museum guides may have had to improve visitor experience, considering both the technical aspects and visitors’ perception. The results of the experiments performed showed the preference for the exhibit visual recognition methods (53% of people prefer them whereas only 14% prefer QR codes, 23% prefer keypads and 10% is undecided). Ease of use, enjoyability, and distance were the cause of this preference. However, authors also concluded that there was a need for further improvement on both the solution performance and the devices used for such task.

In addition, most museum guide applications have been designed for mobile phones and smart glasses [42,43,44]. However, both approaches presented some disadvantages. In the case of mobile phones, visitors need to explicitly run the application, focus on the painting with the phone camera, and then check the information. Since these actions involve more effort than simply pressing a number in a traditional keypad, visitors tend to prefer the traditional option (this is similar to what it occurs with the QR code approach). If the mobile phone is carried around the neck to alleviate the discomfort, problems with the perspective arise. For example, if the user turns the head to the side, the camera of such devices continues to capture the frontal image instead of capturing the image from the scene that the user is actually observing. This calls for methods and devices in which the camera is positioned close to the user eyes. Smart glasses have been also considered, however, they present issues for users that already wear prescription glasses; furthermore, smart glasses often suffer from overheating. In all cases, cost and battery life continue to pose a big challenge.

Another approach that is being currently studied is the use of convolutional neural networks to recognize objects and, in this particular case, artworks. In [45] the authors analyzed several CNN architectures suitable for artwork recognition on a mobile device, proposing also a configurable multi-scale network-in-networks that allows one to adjust the trade-off between accuracy and computational time.

In [46,47] a similar hands-free and attention mechanism approach as the one proposed in this article is shown using a CNN for artwork recognition, but using a NVIDIA® Shield™ K1 tablet as the wearable device. However, this combination of hardware and method produced a huge power consumption, reducing autonomy to non-useful levels (this will be explained later in Section 7.3).

Finally, commercially-existing solutions for automatic museum audio guides do not fulfil the list of desired functionalities such as automatic recognition, hands-free, or low power consumption. Table 1 summarizes the advantages and disadvantages of the existing solutions.

In this paper we propose a solution that uses the wearable device to automatically recognize the paintings from the image captured by a camera, and provides the user with the associated information as audio feedback. Unlike other devices, the battery consumption will allow the device to be used for a day span. Moreover, it will not have problems capturing the image from the user’s point of view and will reduce user interaction to a minimum, increasing the visit experience to a maximum. The visit is thus transformed into a holistic experience allowing both knowledge transfer/interpretation and an undisturbed museum visit.

## 3. Proposed Hands-Free Museum Audio Guide Hardware

In our novel wearable automatic “museum audio guide” for museums, an audio headset is equipped with the highly-efficient EoT board for computer vision and a small camera. The EoT board runs image analysis software that is able to recognize paintings while the user is looking at them. The painting recognition module is running continuously, but does not consume much power thanks to the dedicated hardware module and the efficient implementation. Thus, the developed camera-equipped headset can be used for an entire day without the need to change the batteries.

On top of the painting recognition module, the control application has been implemented, which allows for a specific behavior upon recognition of the painting. The current implementation contemplates two scenarios. In the first scenario the user receives a short audio notification indicating that additional information about the recognized painting is available, without requiring an additional companion app or device. Then, the user may decide to play the audio information by pressing a button on the headset. The second scenario extends the visitor experience by a companion mobile application that offers additional multimedia information and interactivity.

The proposed hands-free audio guide is based on the aforementioned EoT platform designed in the Eyes of Things project in which the authors were involved [22]. The board was designed by Intel Movidius using its Myriad2 MA2450 processor taken into account the component selection performed by the project consortium to achieve the four objectives: Size, cost, flexibility, and power consumption.

### 3.1. EoT Device

**Figure 1:**
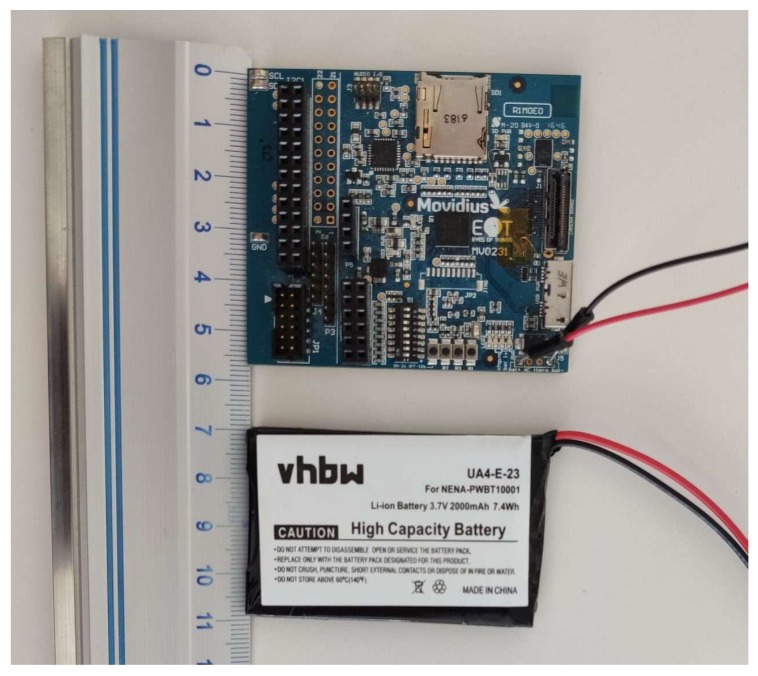
EoT platform.

**Figure 2:**
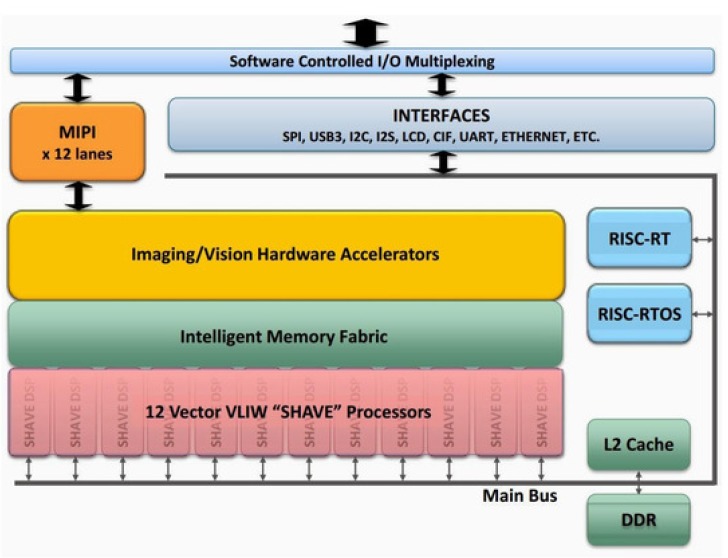
Myriad 2 SoC architecture.

The EoT form-factor device is an eight-layer high density PCB (Printed Circuit Board), that has been customly designed to reduce cost and size and to manage power efficiently (Figure 1). The low-power Myriad2 MA2450 vision processing unit (VPU) is used for all processing and monitoring. Myriad 2 is a heterogeneous multicore VPU composed of twelve propietary very long instruction word (VLIW) processors called SHAVEs, two 32-bit reduced instruction set computer (RISC) processors (LeonOS and LeonRT), and a hardware acceleration pipeline for computer vision tasks (Figure 2). The chip has a 2MB on-chip memory and an internal bandwidth of 400 GB per second apart from a 512MB DDR3 DRAM. It was designed to operate at 0.9V for nominal 600MHz operation. In addition, parts of the processor can be switched off thanks to the provided power-islands. All the built-in core processors with video accelerators and pipeline hardware achieve 1000 FP16 GFLOPs at 600 mW.

The system performs the always-on vision processing tasks thanks to the low-power included camera sensors: AMS International AG/Awaiba NanEye2D and NanEyeRS, Himax HM01B0 along with the Sony MIPI IMX208 high resolution sensor (Table 2).

**Figure 3:**
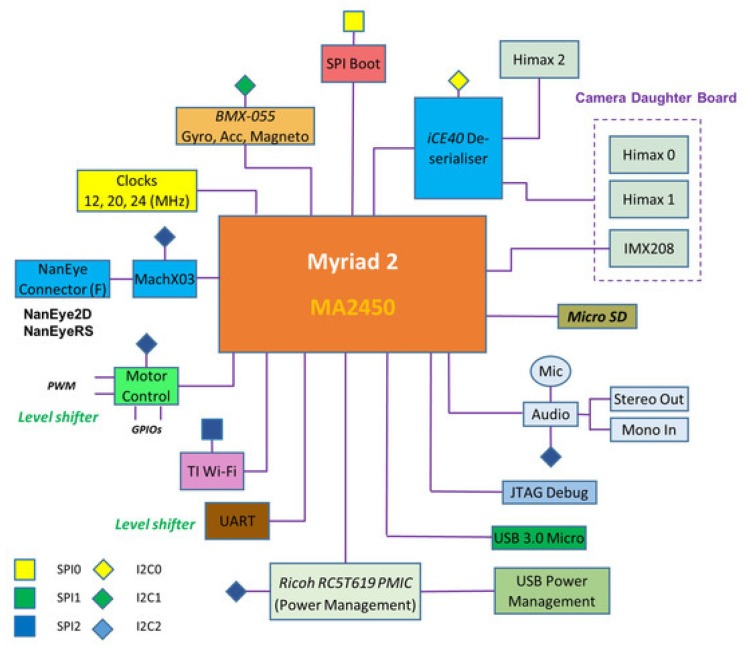
EoT block diagram.

The decisions made based on the extracted information from the sensors data can be sent to other devices or to the cloud through the integrated low-power Wi-Fi module. Audio signals for prompting and warning are allowed in personal assistant cases through a full onboard audio codec (Figure 3). In addition, integrated level shifters expose motor control pins. The EoT device can run for up to 24 hours from a fully charged Li-Po battery by combining the Myriad2 VPU, low-power sensors and an energy efficient component selection during the board design. Finally, USB and micro-SD encrypted functions allow fast and secure data logging. A detailed description of the EoT board can be found in [23].

### 3.2. Headset Design

**Figure 4:**
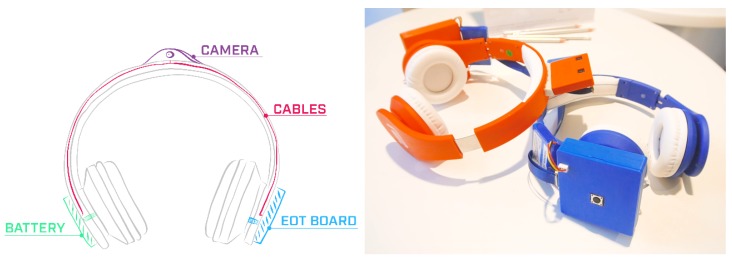
Museum Guide Headset concept draft and prototypes.

A wearable headset prototype for a real demonstration and piloting context has been developed (Figure 4). For this, 3D-printing designs were made for cases for the EoT board, battery and camera. These cases were then printed and attached to commercial headphones.

The EoT board with the camera and the battery add only 120 gr to the headset which weights 300 g. Since the headset weight usually ranges between 100 g and 400 g, the total weight of the prototype is not a problem for the user [53]. Furthermore, the debug parts of the EoT board can be permanently removed to reduce the final weight. A final, market-ready product will be expected to be a fully redesigned aesthetic headset, which can integrate other advanced technologies such as bone conduction earphones.

## 4. System Design

The first part of the design process consisted of clearly identifying the different use case scenarios that were to be covered. Two different scenarios were highlighted depending on the use or not of a companion device. Although the proposed solution will be able to work in standalone mode, it should be able to operate paired with a companion device to provide additional information if the user requires it. Thus, the solution should be able to operate in both cases and it will be the user who will decide according to his/her preferences. Having this in mind, the identified scenarios are a “hands-free museum guide” and a “multimedia museum guide”.

**Figure 5:**
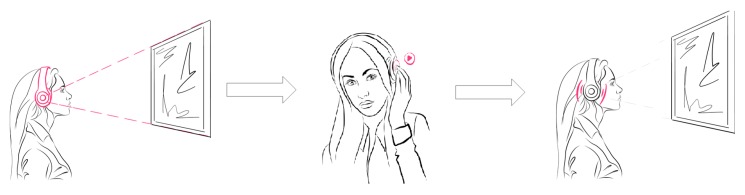
Hands-free museum guide scenario.

The hands-free museum guide is based on the classical audio guide use case, and therefore it is restricted to audio interpretation only (Figure 5). Several steps were identified in this scenario:

1. Visitor enters the exhibition and receives EoT enhanced headset;
2. Visitor explores the exhibition;
3. Visitor is notified about available audio interpretation from the recognized exhibits;
4. Visitor listens to audio interpretation and continues exploring the exhibition.

On the other hand, in the multimedia museum guide the user can use a smartphone or a tablet to retrieve visual information about the recognized exhibits. The steps identified in this scenario are:

1. Visitor enters the exhibition and receives the enhanced headset which connects to an application on the visitor’s smartphone;
2. Visitor explores the exhibition;
3. System detects available exhibits;
4. Visitor receives rich content and/or performs interaction.

**Figure 6:**
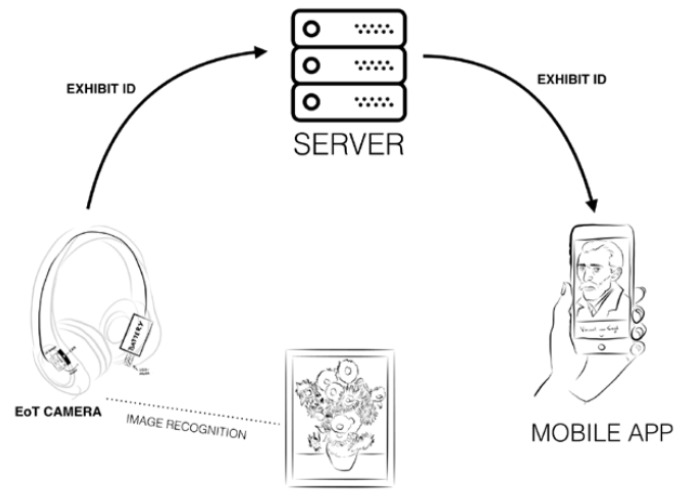
Multimedia museum guide scenario.

In addition, this scenario involves a communication mechanism between the headset and the companion device (Figure 6). The EoT board includes a low-powered WiFi module that can be used to perform it. However, the software selected to implement the communication is also crucial. In this case, the message queue telemetry (MQTT) transport protocol was selected [54]. MQTT is a lightweight transmission protocol that was designed for small sensors and mobile devices. It has demonstrated to be much more efficient than other protocols such as HTTPS [55].

The information retrieved by the companion device can be stored in the device itself or in a server. Whereas the former makes the content available without downloading additional data, the later facilitates data updates without reinstalling the application or modifying the stored files. Furthermore, having the information about the exhibition centralized prevents redundancies or inconsistencies and facilitates the operators work. Therefore, the development of a backend application hosted in the museum’s server is the proposed approach.

To allow the museum operators to edit the content the companion device can access, another application is required. Since this application does not require to be executed in a mobile device, it can be developed as a web page that access the server and retrieve/edit the information.

Since EoT and companion devices needs to be paired, a mechanism to accomplish it is needed. Common approaches involves NFC and Bluetooth technologies [56]. However, due to the power restrictions, the EoT board does not include them. An alternative is to use a method consisting on pairing devices using QR codes. This approach is commonly used to pair devices through WiFi, e.g., to pair WhatsApp and WhatsApp Web. Recognizing QR codes is not complex and can be done in the EoT device.

**Figure 7:**
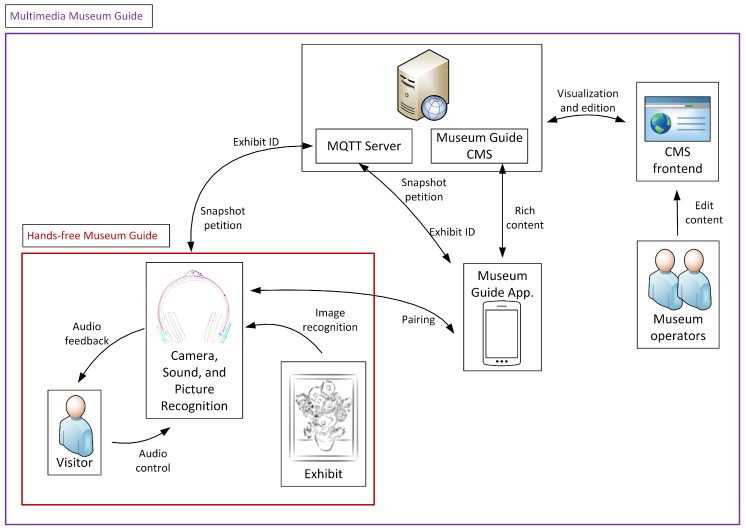
Architecture of the complete system.

Figure 7 depicts the global overview of the proposed system considering the two use case scenarios and all the applications and devices involved.

Finally, the painting recognition module is based on keypoint extraction and classification in the spirit of the “matching by classifying" paradigm [57].

## 5. Applications

The software developed for the museum audio guide consists of several applications:

- EoT-based headset application;
- Museum Guide CMS;
- Museum Guide mobile application;
- CMS frontend for museum operators.

The software running on the EoT-based headset consists of four modules: Image acquisition, painting recognition, network communication, and application control. The functionalities of each module are:

1. The camera module provides control functionality for the camera. It provides frame rate control, auto-exposure (AE) and automatic gain control (AGC). The raw frames from the camera are pre-processed by denoising them, eliminating dead pixels, applying lens shading correction, gathering statistics for AE and AGC as well as applying gamma correction.
2. The painting recognition module takes care of recognising paintings based on their visual appearance (see Section 6).
3. The network communication module handles communication with the MQTT. It sends out notifications for recognized paintings and receives requests to take snapshots of what the camera sees.
4. The control module contains the code that ties the functionalities of the other modules together and adds other functionalities. It handles the pairing of a companion device, signaling that a new painting was recognized and handling input from the push button to control audio pause and playback. Two implementation variants of this module exist: One for the hands-free scenario and one for the multimedia museum guide scenario. Pairing and network communication are only done in the multimedia version. Otherwise, the functionality is the same.

Depending on the use case scenario, different devices and applications are involved. The following sections explain the implementation details for both the hands-free museum guide and the multimedia museum guide.

### 5.1. Scenario 1—Hands-Free Museum Guide

**Figure 8:**
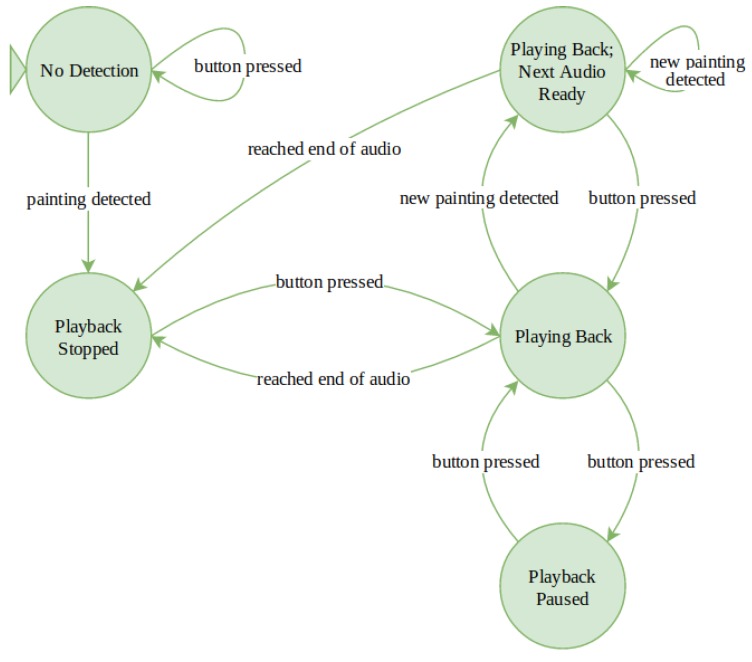
Hands-free Museum Guide state diagram.

In this scenario the headset equipped with the the EoT board and the camera works in a standalone mode, without the need for a companion device. Figure 8 depicts the state diagram of this scenario. The workflow is as follows:

1. Visitor enters the exhibition and receives a headset which is ready to use. No other devices will be handed out. The visitor only has to put on the headphones and start exploring the exhibition.
2. Visitor explores the exhibition. Now the visit starts as like any other usual visit. No devices are carried around the neck or in the hands of the visitor. The visitor is only equipped with headphones. During the visit, the EoT device permanently tracks the view of the visitor. “Always-on” camera and image recognition algorithms permanently try to detect exhibits for which content is available. This works in the background without disturbing the visitor. And even if an exhibit is positively detected, the system waits for a specific time before the visitor is notified. The reason for that is that we generally want to provide the visitor with information about an exhibit only in a moment where there is a very high probability that the visitor is actually focusing on the respective exhibit, i.e., is open for additional knowledge about that very exhibit. Otherwise, the visitor would be notified too often. For this reason a time threshold has been implemented.
3. Visitor is notified about available audio interpretation. After a positive image recognition match the device only notifies the visitor about the fact that content is available with an unobtrusive and discrete sound signal. Thus, playback is not started automatically until only after user action. The main reason for this is that automatic playback may be too intervening and disturbing. It would directly force the visitor to react (to stop the playback or take off the headphones) if he/she does not want playback at that time.
4. Visitor listens to audio interpretation. This is where the only user interaction with the device takes place. As simplicity is key, starting playback is triggered simply by pressing the (one and only) user button on the enhanced headset. This starts the audio playback. By pressing the button again, playback is paused. To continue a paused playback, the button has to be pushed again. This seems to be the most intuitive way for user interaction in order to cover the minimum user control of play and pause. Image recognition continues during listening to active playback. Following our observations, many visitors continue listening to an exhibit audio while moving to other exhibits. Therefore, recognition continues and triggers the detection signal while listening to a previously detected exhibit audio. If a user then taps the play button the currently played audio is stopped and the new one is played.

### 5.2. Scenario 2—Multimedia Museum Guide

**Figure 9:**
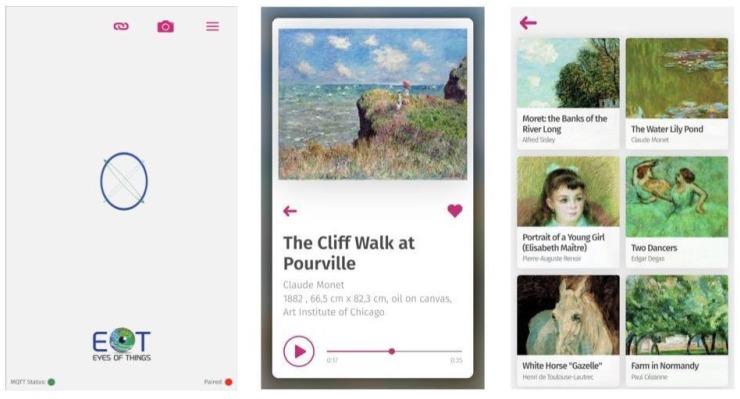
Views of the companion application after pairing.

In the multimedia museum guide scenario the headset communicates with a hand-held companion device (smartphone or tablet). Thus, the user can obtain more multimedia information about the exhibition. For that, a full-fledged multimedia museum application which connects to the museum guide device has been developed. It provides a unique opportunity for museums to provide their museum guide visitors with multimedia and interactive features. The museum guiding application enables users to receive additional content like photos, videos and text about the museum exhibits under demand. It also allows to add exhibits to a favorite list which preserves a personal memory of the museum visit. After pairing with a museum device, the application receives positive image recognition via MQTT and triggers respective actions like a detail view of the exhibit with extended, multimedia content (Figure 9).

1. The visitor enters the exhibition and receives the enhanced headset which connects to an application on the visitor’s smartphone. The “visitor application” can be downloaded on smartphones/tablets or alternatively, visitors may receive a mobile touch device from the museum which is pre-deployed with the application and preconnected to the device. An easy to use pairing mode allows one to quickly connect any smartphone to the museum guide device via the application using the MQTT protocol combined with QR code recognition.
2. The visitor explores the exhibition. The visitor puts on the headset and starts the museum visit. During the visit the device permanently tracks the view of the visitor. Image recognition algorithms automatically detect exhibits for which content is available.
3. The system detects available exhibits. After an image recognition match (combined with a specific threshold time), the headset device notifies the mobile application via MQTT that a registered exhibit has been detected.
4. The visitor receives rich content and/or performs interaction. The visitor is notified via audio signal on the headphones and in the mobile application, and then is provided with rich content, for example, the biography of the artist, the “making of” of the image, other related artworks, etc. The user is allowed to interactively zoom and pinch through high-resolution images or consume videos. This makes the system a full developed multimedia museum guiding system.

**Figure 10:**
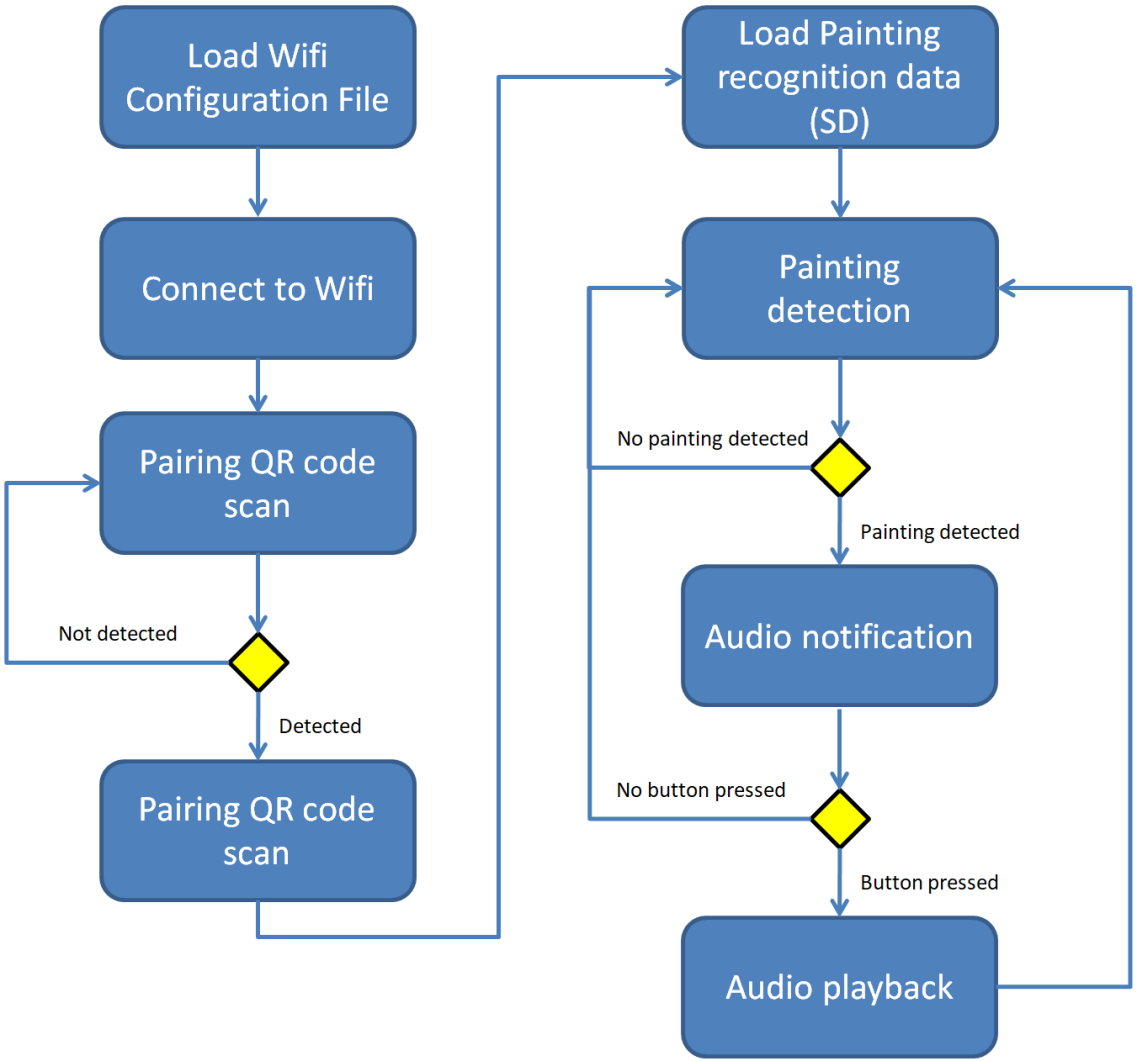
Flow diagram of the headset application in the multimedia museum guide scenario.

Figure 10 depicts the flow diagram of this scenario.

The frontend of the museum guide application is implemented as a mobile web-application. By leveraging standard web technologies (JavaScript, HTML5 and CSS3) the application can be accessed using any mobile browser, which makes it highly accessible and allows for easy distribution. For preview purposes the museum application can also run in a regular desktop browser. Persisting data (e.g., for storing favorite exhibits) is stored in the user’s browser using localStorage. Audio playback is implemented using HTML5 Audio elements that can be controlled by the user via user interface (UI) elements for play/pause/seeking. The museum application is ready to be packaged as a native mobile application using Apache Cordova. This enables additional distribution through Apple Application Store as well as Google Play Store.

For the application backend the following have been used:

- Tech-Stack: Apache Webserver, PHP 7.0.13, MySQL Database, CodeIgniter PHP-Framework, NodeJS;
- Management and hosting of media files (Images, Audios);
- Management of all exhibits;
- HTTP-based API (JSON-format);
- Caching of files for increased performance.

As mentioned above, the real-time communication between the device and the museum application is realized via MQTT which is a publish/subscribe messaging protocol. Both the headset application and the museum application register as MQTT clients to a central MQTT broker (server). This broker handles the distribution of messages between the clients. The museum application subscribes to one main topic (channel): “Detection". The detection message is broadcasted by the headset application and includes a unique identifier of the recognized exhibit as well as a timestamp. When multiple devices as well as multiple mobile applications are used, the “detection" topic is extended by a session identifier which allows targeting an individual mobile application user. When the museum application receives a detection message, it opens the details of the exhibit.

**Figure 11:**
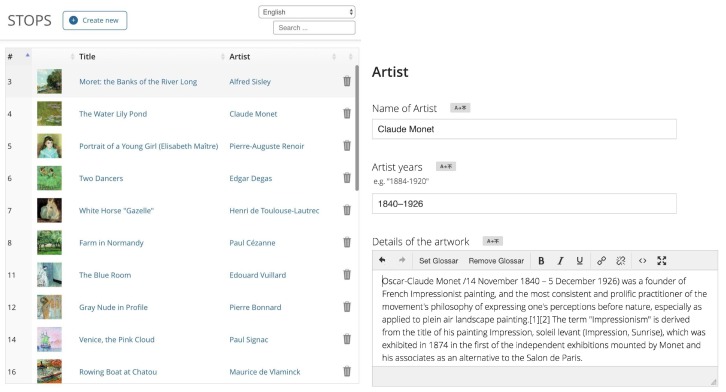
CMS: Exhibit-content and text-editor.

In addition, we have developed a complete museum guide content management system which allows the operator of the system (museum) to add, edit, and delete content of the visitor application (Figure 11). This component completes the system with a web-based, easy-to-use front end for the museum guiding services and is key for a later stage. This system has the following features:

- Via web browser (no additional software needed);
- Easy adding and editing of exhibits;
- Rich text editor for exhibit content;
- Photo/video/audio uploader with:
  - Automatic background conversion;
  - File size reduction mechanisms;
  - In-browser editing/cropping of images;
- File manager.

## 6. Painting Recognition

The painting recognition module is called for each incoming camera frame when the device is in “painting recognition" mode. These modules take a camera image as input and provide a painting ID as output. If no painting has been identified in the current frame, a specific ID “no painting" is returned. The painting recognition pipeline was designed to only use a small number of the available processing cores in order to minimize power consumption. The final EoT-based headset application is able to operate with only the LeonOS, LeonRT and one of the SHAVE core processors. From them, only the SHAVE core was used to recognize the paintings obtaining a frame rate of 10-60 FPs. Since this was more than enough for our museum guide requirements, there was no need to use more cores and increase the power consumption of the application [58]. The painting recognition pipeline is explained below.

### 6.1. Keypoint Extraction

Functionality keypoint extraction takes the camera image as input and extracts FAST keypoints from the image [59]. Each keypoint is identified through its (x,y) coordinates. The selection of this approach is justified by its demonstrated trade-off between robustness and speed [60,61], as one of the main requirements of the prototype is to be able to manage the computation on the embedded device in real-time.

### 6.2. Keypoint Classification

Functionality keypoint classification takes a keypoint position and an image as an input. Classification is based on a random forest consisting of a number of trees [62]. Each single tree takes the keypoint position as input [63]. A tree node consist of a binary brightness test between two specific pixels in the direct neighborhood of the keypoint. According to the result of this test, the point is passed to the next node in the tree. The tree leaves consist of a probability distribution of possible keypoints as learnt in the learning phase. Thus, each keypoint will generate a probability distribution among the possible reference keypoints for each tree. The distributions of all the trees are averaged in order to provide the global probability distribution, from which the maximum is taken. If this maximum is above a threshold, the keypoint is classified as a known keypoint. Repeating this process for all the keypoints in the current frame allows for counting the number of keypoints in the current frame that correspond to a given painting. In addition to its robustness and reduced computational time, this approach is much faster in training and testing than traditional classifiers, thus reducing set up time [64].

### 6.3. Geometric Refinement

In order to avoid false positives, the found keypoints are tested for correct geometric alignment. To this aim, the function attempts to recover a homography between the input image and the reference painting, using a robust method based on the RANSAC paradigm [65]. This method is able to separate inliers (keypoints participating to a global geometric transformation) from outliers (false positive). Thanks to the geometric refinement, the statistics on the number of found keypoints is much more robust.

### 6.4. Model Training

**Figure 12:**
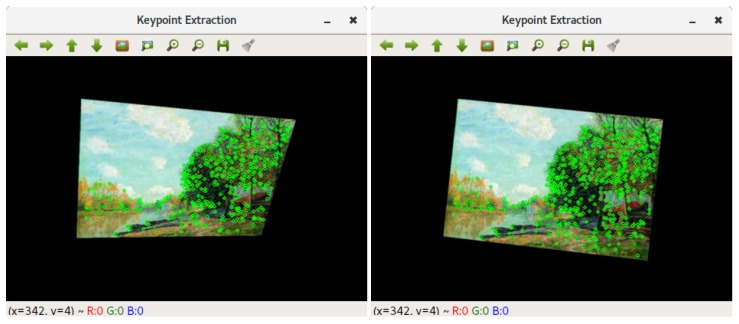
Rendering of a painting in two random perspectives. Detected keypoints are marked with green circles.

The software to train the painting recognition consists of two separate applications. The first one analyzes an image of a painting and extracts suitable keypoint locations. The suitability of a keypoint is defined by how reliably it can be detected from different points of view. The program determines a stochastic approximation to that value by rendering the image in 1,000 random perspectives and counting in how many of those a given keypoint can be detected (Figure 12). The keypoints which have been found and scored this way are ordered by their quality and stored in a file. The second application reads that keypoint file as well as the image and trains a random forest to recognize the keypoints of that painting. Training is also performed in a stochastic manner by again rendering the painting in random perspectives. For a given keypoint a sample of the keypoints appearance is drawn from each random view and fed to the training process of the random forest. The resulting forest is then serialized into a file, which can be read from a microSD card by the museum guide software running on the EoT board.

## 7. Results

To validate the system different evaluation methodologies were used depending on the objective. The painting recognition module, user experience, computational time and power consumption of the system were analyzed in detail.

### 7.1. Painting Recognition Results

To analyze the performance of the proposed method, a synthetic evaluation has been performed using 19 art pieces present in the museum collection of the test pilot. This experiment evaluates the number of keypoints that were correctly classified on average for each painting, while the painting is being synthetically rotated.

The limits for the rotations in the synthetic evaluation are, considering that all the values are with respect to the camera looking at the center of the painting and using intervals of 1 degree, as follows:

- Pitch: −5 degrees to +30 degrees (looking from above or below);
- Yaw: −50 degrees to +50 degrees (looking from left or right);
- Roll: −10 degrees to +10 degrees (tilting the head/camera by up to 10 degrees).

Each painting produces 155 rotated images. Table 3 shows the results of the experiment. Each row contains the information of a particular painting of the dataset: (1) Identifier, (2) the depth of the trees in the forest, (3) the number of trees in the forest, (4) the average number of inliers after applying random sample consensus (RANSAC) and (5) the average percentage of points correctly classified per painting on the whole set of rotated images.

Both depth and number of trees were adjusted during training. From the results, it is worth noting that, as per the proposed algorithm behavior, not all keypoints need to be correctly classified for a correct detection of a painting. In fact, we saw that the number of inliers required must be above 25 in order to detect a painting. Thus, all paintings were correctly detected in the synthetic test with no false positives.

### 7.2. Test Pilot in Albertina Museum

**Figure 13:**
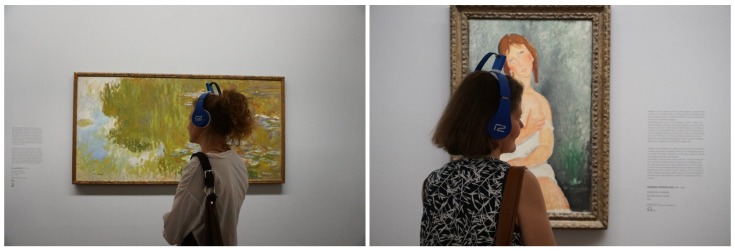
Images taken during the pilot in The Albertina Museum.

The main testing was performed with a piloting phase at a real museum. It was rolled out with one of the worldwide most renowned art museums, The Albertina Museum in Vienna. Within this activity, we prepared two test/piloting events on site during opening hours, invited 13 piloting test users (museum professionals), performed testing days with prototypes, rolled out a survey, did qualitative assessments, did a market potential analysis, and evaluated all results. The process was carried out under the supervision of the ethics board of the EoT project. The museum collections, exhibitions and topics to find the most suitable piloting space for the museum guide pilot testing were analyzed. We decided to go for one of the permanent exhibitions due to the availability of audio content for many exhibits and the good light/space conditions (Figure 13).

The Museum Guiding system was programmed with all data necessary for image recognition at the selected exhibition. We also added the respective audio files for each recognizable painting provided by the museum.

Two desks were set up in the exhibition, one in front of the entrance and another one at the end. Whereas the first desk provided the visitors with the prototypes, the second desk collected/received back the Museum Guide devices and also performed a short interview with the testers as well as provided them with the evaluation questionnaire.

The testing workflow pretty much simulated the designed Museum Guide workflow from the specification phases, i.e., testing visitors arrived at the Museum Guide welcome desk, got a short personal introduction, received the prototype devices and then entered the exhibition. During their visit, the Museum Guide recognizes paintings, giving additional audio storytelling on the artworks due to short notifications and one-button-push for playback. One person from the welcome desk accompanied the testing visitors in order to take general observation notes on user behavior as well as to give assistance if needed.

In order to evaluate the piloting test runs, a multi-chapter questionnaire which queried (1) design, handling and functionality of the museum guide prototype as well as (2) the market potential of a fully developed museum guide was designed. The questionnaire contains a total of 40 different questions and topics. Question types were designed as a variation of likert scale assessments, single-/multiple-choice questions, and open questions. It also contains a demographic section for specifying the context of the tester.

The other evaluation method used was the so-called “thinking aloud", i.e., a participative observation method where a person escorted the participant during their exhibition walkthrough with a voice recorder. The participant was asked to articulate his/her thoughts during the test run giving contextual insights on positive and negative opinions and experiences.

**Figure 14:**
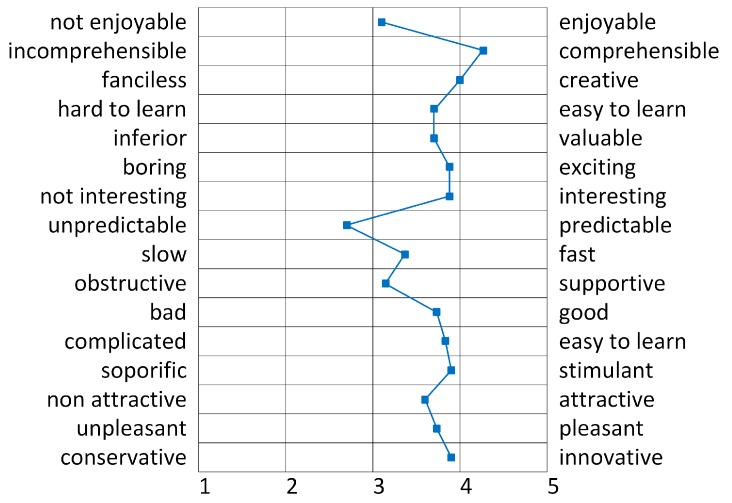
Evaluation results of bipolar assessments.

At first, testing visitors had to spontaneously rate the prototype between 1 (very good) and 5 (very bad) on bipolar concepts like “creative–fanciless", “boring–exciting", or “fast–slow". This is a well-known method to get an intuitive assessment of the subjective impression on the tested service. The positive peaks in Figure 14 show that users especially valued the experience as creative and stimulant as well as “comprehensible” and “easy”. This indicates that the system was keeping its promises on its main benefit which was simplicity of use due to minimalistic user interaction. Image recognition made the handling very easy because of automatic detection and the fact that, contrary to available guides, the hands were free during visit. However, the factor “enjoyable" did still not perform very well which may indicate that, as the concept itself was appreciated, the real experience did not work perfectly due to the image recognition performance. The latter seemed not to work perfectly in satisfying end users needs. This correlated with our observations on the field and “thinking aloud" observations (as well as open questions from the survey) which showed that most users were puzzled by the fact that recognition varied from exhibit to exhibit and depended on distance and angle towards the painting. It is worth noting that the main cause of that unreliability was that, as the device was locally manufactured, changes of the camera direction caused by the movement of the user or even its accommodation may have caused the algorithm to miss the keypoints due to the impossibility of framing the painting. To improve performance, a camera with a wider angle lens or a stabilization mechanism would be advisable.

**Figure 15:**
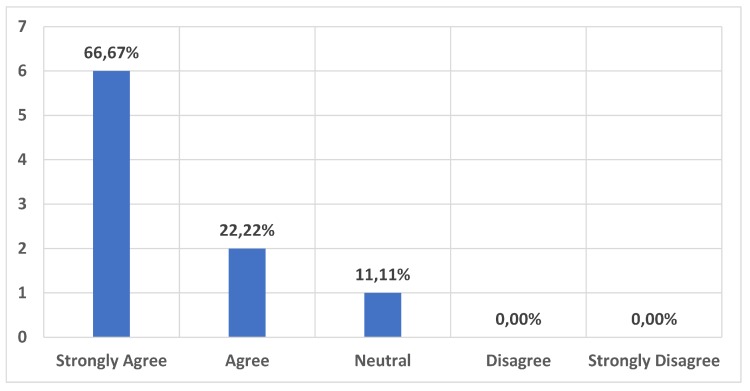
Complexity of the audio content. The audio content was easily comprehensible and not too complicated for the average museum visitor.

**Figure 16:**
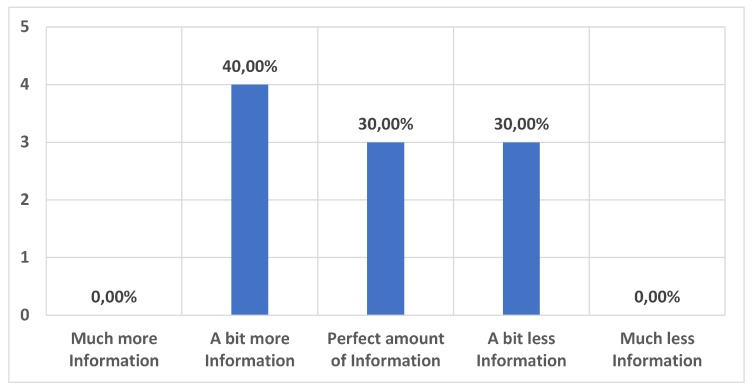
Suitability of the audio length. How appropriate was the length of the audio stories? Visitors would rather have had...

We also assessed the quality of the content as well as the length of provided audio content. The results can be found in Figure 15 and Figure 16. These results show that the amount of information and length of audios were pretty balanced and suitable for a museum guide environment.

**Figure 17:**
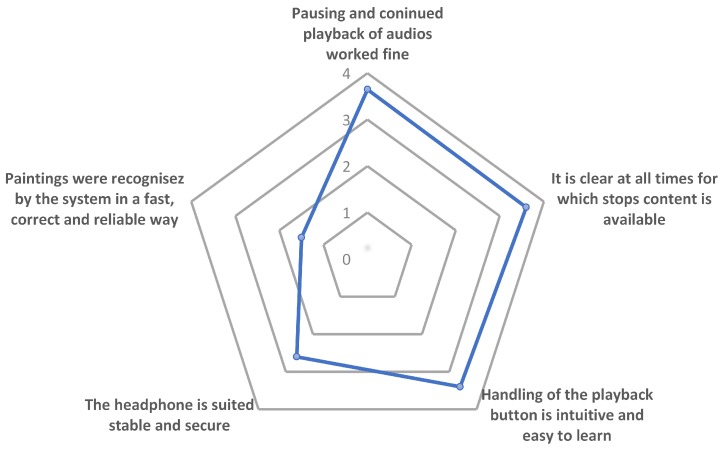
Assessment of user experience to the question. How do you rate the handling of the tested prototype?

In addition, we tried to assess the actual handling, operations, and user experience of the museum guide prototype and how it worked for the participants. We asked detailed questions about how the image recognition performed, how the visit was experienced, and whether the detection workflow and interaction with the device was intuitive or complicated. Note that, in the visualization depicted in Figure 17, users were more unsatisfied the farther the value for each category iwa away from the center. Hence, the results clearly show the main problems of using the museum guide prototype in image recognition performance and headphone stability. The latter is due to the prototype nature of the assembly and will be brought to a next level in further product improvements. For the image recognition problem, we think that a wide-angle vision would be beneficial.

On the other hand, the system seemed to perform reliably concerning the user experience workflows and general usability. The latter is extremely important as the Museum Guide is a fully automatic unit where it is crucial that users know what the system is doing and why. This seems to be the case as well as results show that handling the button interaction worked well and intuitively.

**Figure 18:**
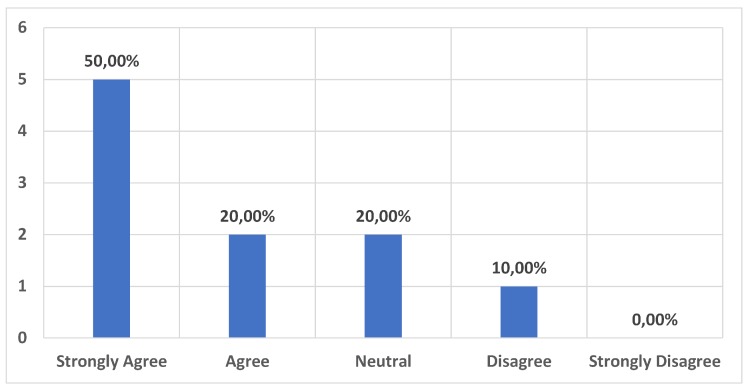
Functional results. A full functional Museum Guide would be a good substitution for the usual audio guide.

As the main competing existing service for the museum guide is the classical audioguide, it was important to understand the potential of the envisioned product in substituting the available, classical, audioguide product existing in many museums. The results shown in Figure 18 strongly indicate that there is a high potential in taking over the existing audio guide market, as 70% are on the positive side and only 10% on the negative side.

### 7.3. Processing Time and Power Consumption

The time required by the the painting recognition module was measured. It is able to work at 10–60 FPS with a recognition time between 16 and 100 milliseconds.

Power consumption was analyzed in two settings: (1) During the painting recognition phase and (2) during audio playback. The power draw during painting recognition was 1.15 W. Audio playback additionally consumes 0.2 W which leads to a total power draw of 1.35 W. Compared with the typical power draw of an Nvidia® Jetson™ TX1 of around 8–10 W under normal computation loads [66], this represents a minimum improvement of six times the operational time with the same battery.

The current prototype device uses a 7.59 Wh battery (≈2000mAh). At full charge this translates to 5:30 h to 6:30 h of operation based on the usage profile. For example, if the audio is playing 80% of the time the battery lasts for approximately 5:45 h. We note that a more powerful battery of 4000 mAh weighing 95 g could be also used, and this would double the battery time to 11–13 h, which will make the device available for the museum opening hours, requiring recharges only during the night.

## 8. Conclusions

The results presented in this article demonstrate the potential of replacing traditional museum audioguides with the proposed system. The complete system was thoroughly evaluated under real conditions. Moreover, the pilot test took place during the opening hours of the museum. Since two different scenarios have been considered, the system is configurable according to what each visitor demands. This fact, and the wearable hands-free nature of the system, are the main reasons why the user experience was satisfactory.

Moreover, the power consumption performance of the proposed device and implementation allows the museum to only charge the device outside of opening hours, as it is able to function for a minimum of 5:30 hours in the most demanding scenario without charging.

Finally, based on this study, we also conclude that the painting recognition module should be improved in further work, since it is the weakest point of the system. This might be done by replacing the camera by another one with a wider view angle, or by improving the physical management of the camera using mechanical stabilization, as the main problem of the current prototype is that variations in the way the user wears the device produces significant changes on the image or impedes capturing the painting at all.

## Figures and Tables

**Table 1:** Comparison of existing solutions.

Application	Method	Advantages	Disadvantages
Audio Guide Crystalsound [48]	GPS positioning	Low consumption and size. Hands-free.	Indoor positioning not included.
Locatify Museum Guide [49]	Mobile phone app. Positioning	Outdoor and indoor positioning.	Power consumption. The user has to select the information from a list because several art pieces are close. Not hands-free.
Copernicus Guide [50]	Mobile phone app. Positioning	Tours are initially downloaded and are available offline. Outdoor and indoor positioning.	Power consumption. Not hands-free.
xamoom Museum Guide [51]	Mobile phone app. QR/NFC scanner	Easy to use	Power consumption. Not fully automatic since the user has to scan the QR.
Orpheo Touch Multimedia Guide [52]	GPS + Camera	Augmented Reality	Power consumption. Not hands-free.

**Table 2:** EoT supported cameras.

Camera	Resolution	FPS	Consumption
AMS International AG/Awaiba NanEye2D	250×250 pixels	60 FPS	5 mW
AMS International AG/Awaiba NanEyeRS	680×680 pixels	50 FPS	13 mW
Himax HM01B0	320×320 pixels	30 FPS	<2 mW
Himax HM01B0	160×120 pixels	30 FPS	1.1 mW
Sony MIPI IMX208	1080×768 pixels	30 FPS	66 mW

**Table 3:** Experiment results. ID is the painting identifier, Depth and Trees are the depth of the trees and the number of trees in the forest, Inliers represents the average number of inliers after applying RANSAC and Classification is the average percentage of inliers correctly classified.

ID	Depth	Trees	Inliers	Classification
1	8	16	85.8	89.08%
2	8	16	49.3	95.26 %
3	8	16	54.2	93.71%
4	8	16	84.7	96.68%
5	8	16	92.9	92.72%
6	8	16	94.9	77.12%
7	8	16	92.1	90.18%
8	8	16	62.3	75.18%
9	8	16	81.5	85.18%
10	8	20	102	76.86%
11	8	20	112	80.35%
12	8	20	93.3	76.83%
13	8	16	103	86.12%
14	8	16	112.3	86.011%
15	8	16	49.5	83.4%
16	8	16	106	81.99%
17	8	16	79.1	85.22%
18	8	16	98.2	81.39%
19	8	16	125	83.74%
